# Colony aging affects the reproductive performance of Swiss Webster females used as recipients for embryo transfer

**DOI:** 10.1590/1984-3143-AR2020-0524

**Published:** 2020-11-24

**Authors:** Geraldine Schlapp, Maria Noel Meikle, Cecilia Silva, Gabriel Fernandez-Graña, Alejo Menchaca, Martina Crispo

**Affiliations:** 1 Transgenic and Experimental Animal Unit, Institut Pasteur de Montevideo, Montevideo, Uruguay; 2 Servicio Médico Integral, Montevideo, Uruguay; 3 Instituto de Reproducción Animal Uruguay, Fundación IRAUy, Montevideo, Uruguay

**Keywords:** pre-implantation embryos, pregnancy failure, pseudopregnant females, foster, cannibalism

## Abstract

The objective was to evaluate the influence of colony aging in a Swiss Webster (SW) outbred stock used as recipients for embryo transfer. In the first study, a retrospective analysis was performed throughout several generations during a 38-month period in 2,398 embryos transferred to 108 SW recipients. A decrease in the percentage of live pups from transferred embryos was found at the end of the period. Impairment occurred due to the incidence of maternal cannibalism that increased from 0% to 67-100% (P<0.05), while pregnancy rate (pregnant/transferred recipients) and number of pups per delivered female were not affected throughout the period (P=NS). A following study was carried out to compare the reproductive performance of SW stock vs. B6D2F1 hybrid females in a 5-year interval. The study was conducted on a total of 893 embryos transferred to 40 females (20 SW and 20 B6D2F1) in Year #1, and 514 embryos transferred to 30 females (15 SW and 15 B6D2F1) in Year #5. No cases of maternal cannibalism were found on Year #1 in any of the strains (0/10 and 0/10). However, an incidence of 44,4% (4/9) was seen on Year #5 for SW, while for B6D2F1 the incidence was 0% (0/12) (P<0.05). Further examination of the uterus showed endometrial cysts and abnormal implantation sites in SW on Year #5 but not in B6D2F1 females. In conclusion, this study reports an impairment of the reproductive performance of an early aged SW outbred stock colony mainly due to the occurrence of maternal cannibalism. This finding has important implications for embryo transfer programs conducted in mouse facilities.

## Introduction

Embryo transfer (ET) is an indispensable tool used in mouse facilities, with several applications in biomedical research. It is essential for the generation of genetically modified mice and as a component of assisted reproductive technologies to overcome fertility problems in mutant animals (Kolbe et al., 2012). Moreover, ET is the first choice to perform embryo rederivation to clean mouse strains from pathogen agents before entering into a barrier housing facility (Sztein et al., 2011; Van Keuren and Saunders, 2004). For this purpose, embryos are thoroughly washed in specific media as recommended by the International Embryo Technology Society (Stringfellow et al., 2007) and transferred into a “clean” recipient female (Sztein et al., 2011). Rederivation by ET can eliminate murine pathogenic virus, *Helicobacter* species, ectoparasites and endoparasites (Van Keuren and Saunders, 2004). In addition, ET of cryopreserved embryos or gametes is a very useful tool to revitalize murine lines.

The success of ET depends on three main factors: the quality and stage of the transferred embryos, the technique and surgical skills of the technician, and the recipient females. When the female strain is chosen as recipient for embryo transfer, it should have the traits of both good reproductive performance and good maternal behavior. In general, F1 hybrids such as B6D2F1, B6CBAF1, and certain outbred stocks such as CD1, CF1 and Swiss Webster (SW) are better suited as embryo recipients compared to inbred strains, mainly due to greater hybrid vigor (Sztein et al., 2011). The use of inbred strains as recipients for ET is also feasible and useful for certain types of studies (Lerch et al., 2016; Rose et al., 2012). Success in a core facility often depends on choosing the best strategy related to recipient strains. Although outbred stocks appear as the preferred option mainly due to their pregnancy outcomes, maternal ability, ease to transfer and big size, they require at least 25 breeding pairs to maintain the outbred condition (Jackson Laboratory, 2009). Usually this issue is solved by outsourcing the recipients from commercial vendors in US, Europe and Asia, but for mouse facilities located in other regions it represents a frequent problem and the females are produced in-house with small colonies. How reproductive performance is affected by the aging of the colony in outbred mice strains such as SW is scarcely understood. As an alternative, the use of F1 hybrids like B6D2F1 obtained by crossing two different inbred strains already available in the facility is an easier and affordable approach.

In the current study we evaluated the reproductive performance of a SW colony used as embryo recipients after several generations (38-month period), in terms of pregnancy and birth rates. In addition, the reproductive outcomes of the current SW colony were compared with B6D2F1 females in a 5-year interval. To our knowledge, this is the first study that reports how the reproductive outcomes of a SW outbred stock is affected by aging through many generations when used as recipient females.

## Methods

### Animals and housing

The study was conducted at the specific pathogen free (SPF) animal facility of the Transgenic and Experimental Animal Unit of Institut Pasteur de Montevideo, Uruguay.

This facility houses 10,000 mice with the aim to produce and maintain genetically modified mice by using CRISPR technology, pronuclear microinjection, embryonic stem cell blastocyst injection and lentiviral transgenesis. The facility manages inbred strains including BALB/cJ, C57BL/6J, DBA2/J, Nude/J and more than 50 genetically modified lines, as well as outbred SW mice and Sprague Dawley rats.

All the animals of the study were bred and housed in our facility in individually ventilated cages (IVC-Tecniplast, Milan, Italy) containing chip bedding (Toplit 6, SAFE, Augy, France). The room temperature was 20 ± 1°C, the relative humidity was 40-60% and the light:dark cycles of 14:10 h. Animals were fed autoclaved food (Labdiet 5K67, PMI Nutrition, IN, USA) and provided with autoclaved filtered water, *ad libitum* (Schlapp et al., 2018). Mice were provided with several nesting and enrichment material as paper towels (in every cage change), cardboard and polycarbonate red houses that were provided in an alternate manner. Health monitoring was performed quarterly, by testing sera or blood spots of BALB/c sentinels. The barrier remained pathogen free from our facility list throughout the experiments (Schlapp et al., 2018).

All the animals were housed and handled according to national law 18.611 and international animal care guidelines (National Research Council, 2011). Experimental protocol (permit number #007-18) was opportunely approved by the Institutional Animal Use Ethics Committee.

The study consisted in a retrospective analysis and an experimental study after the detection of a decrease in the reproductive outcomes of the Swiss Webster stock used in the SPF animal facility. SW animals were originally from Charles River laboratories.

### Retrospective analysis: SW performance during a 38-month period

A retrospective analysis was performed using the data registered from SW females used routinely as recipients for embryo transfer during a 38-month period, that were merged every two months for statistical analysis. Data were recorded two years after the colony was established. Evaluated parameters were: pregnancy rate (pregnant/transferred recipients), produced live pups from transferred embryos (in pregnant and total females), number of pups per delivered female, and cannibalism/delivered female.

During the evaluated period the SW females were produced in a breeding colony composed by a population of 25 to 30 mice (2:1 female:male ratio), housed through 11 generations. The breeding scheme to maintain the colony consisted of a HAN-rotation system (Rapp, 1972) as recommended for small size outbred stock colonies (Berry and Linder, 2006). During this period, colony management was conducted under the same conditions in terms of environment, food supplier, animal caretakers, and technicians for embryo production, zygote microinjection and embryo transfer.

The retrospective analysis includes a total of 2,398 embryos that were produced in B6SJLF1 genetic background females. The embryos were transferred to 108 SW recipient females (in average 22 embryos per female) at 0.5 days post coitum (dpc) after zygote pronuclear microinjection for different transgenic projects. Embryo production and zygote transfer were conducted as described below.

### Reproductive outcomes in SW vs. B6D2F1 recipient females

Reproductive outcomes were compared between SW and B6D2F1 recipient females obtained at two different moments in a 5-year interval: a) At the beginning of the retrospective analysis described above (named as Year #1), and b) one to two years after the end of the period evaluated in the retrospective analysis (named as Year #5). During this period the SW colony was maintained by the breeding scheme described above through 18 generations. In the Year #1, a total of 893 zygotes were transferred to 40 recipient females (20 SW and 20 B6D2F1) at 0.5 dpc. The embryos were produced in B6SJLF1 females and subjected to pronuclear microinjection in transgenic projects conducted in our facility. In the experiment conducted in the Year #5, a total of 514 fresh eight-cell or morula stage embryos were transferred to 30 recipient females (15 SW and 15 B6D2F1) recipient females at 0.5 dpc. In this experiment, the embryos were not subjected to microinjection and were transferred soon after recovery from B6D2F1 donor females. In both studies, pregnancy diagnosis was conducted by visual inspection by an experienced animal caretaker two weeks after ET and litter size was recorded seven days after birth in order not to disturb maternal care. The occurrence of cannibalism was defined as the absence of pups in the cage of a pregnant recipient mother.

### Embryo collection

Unless otherwise indicated, chemicals were purchased from Sigma Chemical Company (St Louis, MO, USA). The embryos were produced by ovarian superstimulation in 3-4 or 8 week-old donor females with a single intraperitoneal injection of 5 IU of equine chorionic gonadotrophin (eCG; Novormon, Syntex, Buenos Aires, Argentina) and 5 IU of human chorionic gonadotrophin (hCG; Chorulon, Intervet International B.V., Boxmeer, the Netherlands) 46 h later. Soon after hCG treatment, females were mated with an adult male in a 1:1 ratio. Donor females were euthanized by cervical dislocation at 0.5 (Year #1) or 2.5 dpc (Year #5), oviducts were excised and zygotes and 8-cell embryos were collected by ampulla tear or oviduct flushing, respectively. Viable embryos were cultured in M16 medium microdrops under embryo tested mineral oil, in 5% CO_2_ in air at 37 ºC.

### Embryo transfer

All recipient mothers (SW and B6D2F1) were mated with in-house vasectomized adult males with proven sterility to induce pseudopregnancy, and checked for copulatory plugs the day of ET (0.5 dpc). Embryo transfer was performed 4-5 h after embryo recovery in the case of microinjected zygotes, and 2-3 h after embryo recovery for fresh non-microinjected embryos. Recipient females were anesthetized with a ketamine-xylazine mixture (ketamine 100 mg/kg, Pharmaservice, Ripoll Vet, Montevideo, Uruguay; xylazine 10 mg/kg, Seton 2%; Calier, Montevideo, Uruguay) via ip (Meikle et al., 2018). Surgery was performed under aseptic conditions. In order to provide post-surgical analgesia, each recipient received one dose of tolfenamic acid (sc, 1.0 mg/kg, Tolfedine®, Vetoquinol, Lure, France), soon after the female was anesthetized (Schlapp et al., 2015). Females were placed over a warm pad to avoid hypothermia, both during surgery and recovery. Embryos were loaded into a pulled glass pipette using M2 medium and placed through the infundibulum into both oviducts (Nagy et al., 2003) transferring 15 to 25 fresh embryos per female. Animals were monitored until full recovery from anesthesia, and inspected the day after the surgery.

### Gross pathology and histological analysis of endometrium

Since a great incidence of cannibalism was found in the Year #5, uterine examination was performed in those SW transferred females that did not produce pups (*i.e*., non-pregnant females and females that produced cannibalism). In addition, uterus of females from the same colony that were not subjected to embryo transfer and B6D2F1 females were also examined. For delivered females, necropsy was performed after pup weaning (i.e. 3 weeks), for those that cannibalized their pups, it was done seven days after the expected parturition day. Samples from all the females were subjected to endometrial examination under optical stereoscopy (40X magnification, Nikon SMZ800, Japan). For histology analysis tissue samples were fixed in formalin 10% for 48h at RT, then washed three times in PBS and stored in 70% ethanol at 4°C. Samples were processed, embedded in paraffin and sectioned. Slide sections were deparaffinized, rehydrated in graded ethanol, and stained with hematoxylin and eosin to be evaluated at 100X and 200X magnification (Axio Observer.Z1, Zeiss, Germany).

### Statistics

In the retrospective analysis data were merged every two months to be analyzed by InfoStat software (Di Rienzo et al., 2020). Pearson correlation coefficients were used to describe relationships between each variable. Logistic procedure was initially used to determine if each individual measurement was affected linearly, quadratically or cubically, and it was used to generate the regression model and determine the slope(s) values according to maximum likelihood estimates from each significant continuous order effect. Logistic regression curves were constructed according to the coefficients provided by the interactive data analyses from InfoStat and to the following equation: *y = ax2 + bx + c*.

Comparison between the outcomes found in SW and B6D2F1 females were analyzed by generalized linear mixed models, and data are expressed as percentage of pregnant/transferred recipients (pregnancy rate), live pups/transferred embryos in total females and in pregnant females (birth rate), and mean ± SEM of live pups per pregnant female. Differences were considered significant for a *P* value <0.05, and <0.1 was assumed as a tendency.

## Results

### Retrospective analysis: SW performance during a 38-month period

**Figure 1:**
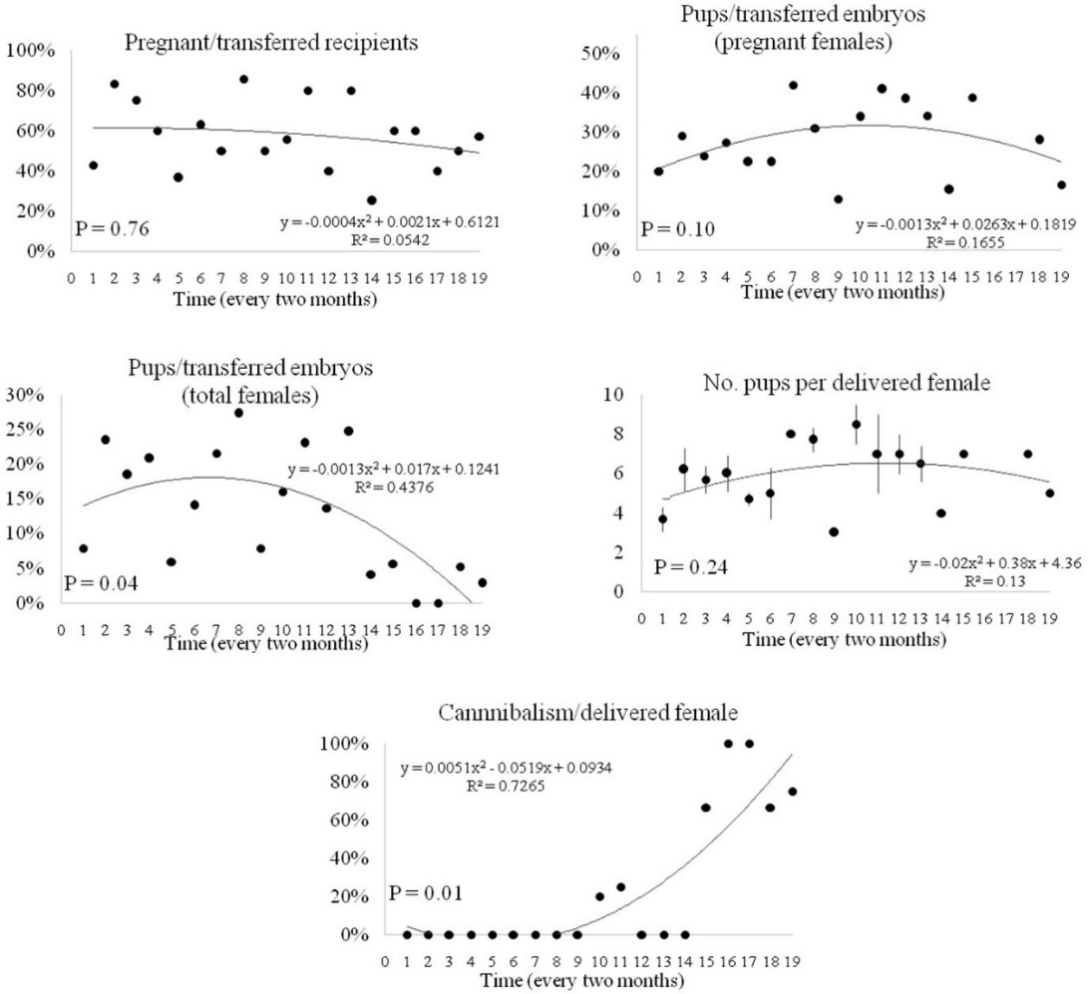
Retrospective analysis of the reproductive performance performed in 108 Swiss Webster mice used as recipient females to receive 2.398 manipulated embryos in a 38-month period. Pearson correlation coefficients were used to describe relationships between each variable and time (merged every two months). Data are shown as percentage or mean ± SEM.

The retrospective analysis showed a significant effect (*P*< 0.05) of the time on different variables of the reproductive performance of SW recipient females. While pregnancy rate (pregnant/transferred recipients) and number of pups per delivered female were not affected in this period, a negative correlation was found at the end of the period in produced live pups from transferred embryos, which was due to a high incidence of maternal cannibalism. The results are shown in Figure 1.

### Reproductive outcomes in SW vs. B6D2F1 recipient females

Pregnancy rate between SW and B6D2F1 recipient females was not different when compared on Year #1 and Year #5. Interestingly, the mean number of pups born per female was lower in SW than B6D2F1 females, and this difference was maintained on Year #1 and Year #5. The same outcomes were found in number of pups from transferred embryos. Of notice, while the occurrence of cannibalism was not found on Year #1 in any recipient strain, it was detected in 44.4% of SW females on Year #5. The results are summarized in Table 1.

**Table 1:** Reproductive outcomes in Swiss Webster and B6D2F1 females subjected to embryo transfer, within a 5-year interval.

	**Year #1**			**Year #5**		
	**SW**	**B6D2F1**	***P* value**	**SW**	**B6D2F1**	***P* value**
Pregnant/transferred recipients	50.0% (10/20)	50.0% (10/20)	NS	60.0% (9/15)	80.0% (12/15)	NS
No. pups/delivered female	6.2 ± 0.8	8.5 ± 0.7	<0.05	5.4 ± 0.7	7.4 ± 0.9	<0.05
No. pups/transferred embryos (pregnant females)	26.1% (62/237)	37.6% (85/226)	<0.05	15.7% (27/172)	39.4% (89/226)	<0.05
No. pups/transferred embryos (total females)	13.5% (62/458)	19.5% (85/435)	<0.05	10.7% (27/252)	34.0% (89/262)	<0.05
Cannibalism/delivered females	0.0% (0/10)	0.0% (0/10)	NS	44.4% (4/9)	0.0% (0/12)	<0.05

NS=not significant; *P* value <0.05 was considered significant.

### Gross pathology and histological analysis of endometrium

**Figure 2:**
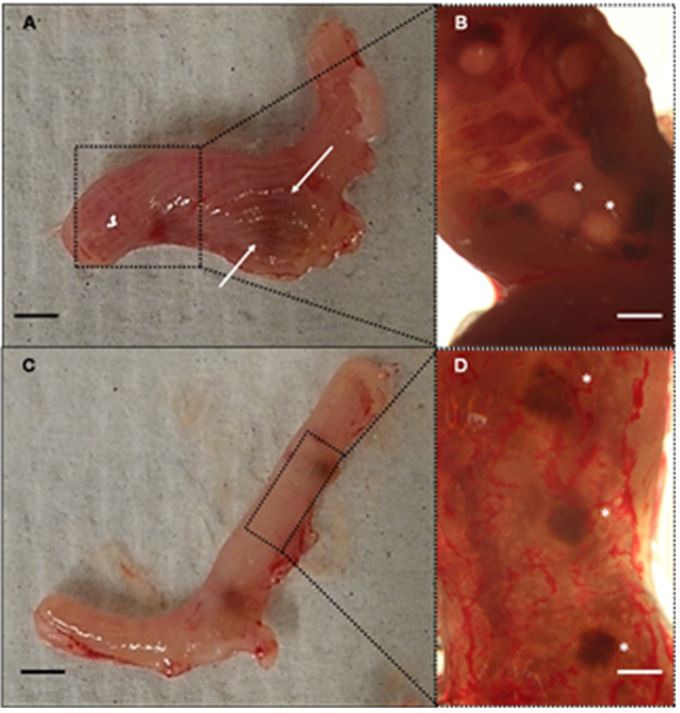
Macroscopic and low magnification observation of recipient female uterus and endometrium. **(A, B)** Uterus from SW female #2, arrows indicate an abnormal implantation site; 40X stereoscopic magnification (dotted box region) of the lumen layer showed endometrial cysts, indicated by asterisks. **(C, D)** Uterus from B6D2F1 female #2; normal implantation sites were seen at 40X in the endometrium (dotted box region), indicated by asterisks. No endometrial cysts were observed. Scale bar = 25 mm (A, C); 0.5 mm (B, D).

Inspection of the endometrium in SW and B6D2F1 female mice is summarized in Table 2. In SW mice cannibalism was found in two females, one of them showing abnormal implantation sites defined as hemorrhagic and bigger than normal (Figure 2A), and endometrial cysts were found at 40x magnification (Figure 2B). These abnormal features were also observed in one of the SW females that received natural mating, even though she was able to deliver eight live and normal pups.

**Table 2:** Postmortem findings after gross examination of the uterus of Swiss Webster and B6D2F1 females.

	**#female**	**Condition**	**Pregnancy**	**No. of live pups** ^*^	**Endometrium findings**
SW	1	ET	Pregnant	0	Normal
	2	ET	Pregnant	0	Abnormal IS; cysts
	3	No mating	Non-pregnant	-	Normal
	4	No mating	Non-pregnant	-	Hemorrhagic
	5	Mating	Pregnant	9	Normal
	6	Mating	Pregnant	8	Abnormal IS; cysts, hemorrhagic
B6D2F1	1	ET	Pregnant	8	Normal
	2	ET	Pregnant	8	Normal
	3	No mating	Non-pregnant	-	Normal

* Absence of pups after delivery in pregnant mice was assumed as cannibalism. ET: embryo transfer; IS: implantation sites.

**Figure 3:**
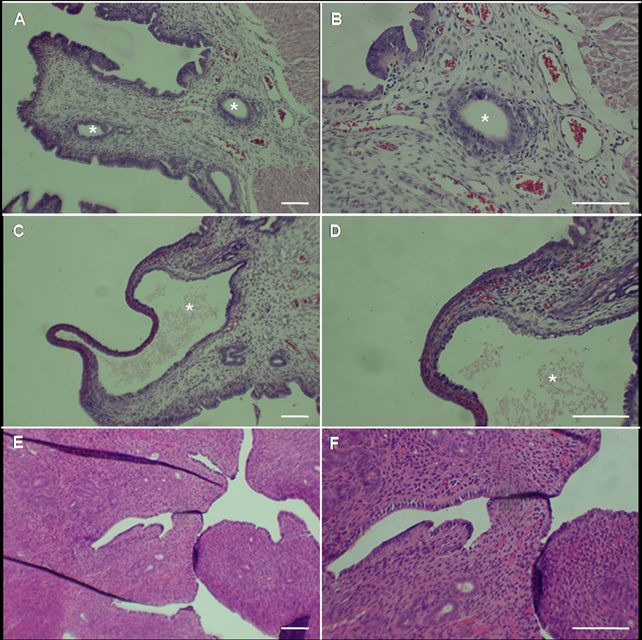
Histological examination of recipient mice endometrium. Observations of H&E slides of SW female #2 endometrium at 200X **(A, C)** and 400X **(B, D)** showed endometrial cysts (asterisks) with pseudostratified epithelium. Normal endometrium was observed in B6D2F1 female #2 **(E, F)**. Scale bar = 100 µm (all images).

Normal implantation sites were observed in all B6D2F1 recipient mothers and neither endometrial cysts nor any other abnormalities were seen (Figure 22D). Histological examination of SW female uterus that presented endometrial cysts showed pseudostratified columnar epithelium and vacuolated cytoplasm, mild hyperplasia and cysts in the endometrial stroma (Figure 3A, B, C and D). Normal endometrium was observed in B6D2F1 uterus (Figure 3E and F).

## Discussion

This study shows that the reproductive performance of SW outbred stock females used as recipients in an embryo transfer program was impaired as the mice colony was aging within a 38-month period. In addition, the decrease in the outcomes of SW mice was confirmed when compared with B6D2F1 hybrid females in two replicates conducted in a 5-year interval. The impairment found in the SW females was related to the occurrence of maternal cannibalism, without affecting pregnancy rate or number of pups born/female in those recipients that produced live offspring.

The recipient mother in which the embryo is placed for pregnancy establishment and maintenance, birth and raising, is a key factor for the success of embryo transfer programs in any species. In mice, several strains, stocks and hybrid females are used as recipients for embryo transfer (Gerlinskaya et al., 2019; Litvinova et al., 2020; Rose et al., 2012) offering the possibility to choose the most appropriate recipient type for each particular project. Outbred stocks and F1 hybrids are often the females of choice to be used as recipients for routine embryo transfer in mouse facilities (Lutz et al., 2012). The SW outbred stock used in this study are one of the most recommended since they have long lifespan, high disease resistance, early fertility, high overall reproductive performance and low neonatal mortality; they are also considerably less expensive (Lutz et al., 2012). In fact, the SW outcomes obtained at the beginning of the period under the retrospective analysis were acceptable within the expected parameters.

The overall reproductive performance evaluated in the retrospective analysis was impaired at the end of the 38-month period. The main finding was a significant fall in live pups produced from transferred embryos, which was due to the occurrence of maternal cannibalism by the recipient mothers. Impairment in reproductive performance in mouse recipients depend on several factors, and genetics or inbreeding of the females should be taken into account. Outbred stocks like SW are defined as a closed population (for at least four generations) of genetically variable animals that are bred to maintain maximum heterozygosity (Festing, 1993a). To maintain this condition, inbreeding may be avoided by using a rotational breeding scheme as the HAN-rotation system used in our facility (Nomura and Yonezawa, 1996). It is well-known that inbreeding produces a decline of the reproductive performance, for instance litter size reduction, also called “inbreeding depression” (Hinrichs et al., 2007). With each filial mating, the proportion of homozygous alleles is increased, often reducing the fitness of the lines (Pritchett and Taft, 2006). In the current study, the fact that impairment of reproductive performance occurred as the period progressed, suggests a possible failure in the maintenance of the colony heterozygosity. In order to study the mouse genetic profile, diverse genetic quality control methods are available, for instance microsatellite markers, skin grafting, DNA fingerprinting, among others. While these methods are suitable to monitor inbred strains, these systems may have little or no value for outbred stocks (Festing, 1993b), which are genetically ill-defined. Moreover, their genetic make-up has been affected by a poor understanding of the breeding schemes used over the time (Chia et al., 2005).

Most commercial mouse suppliers of outbred stocks apply breeding schemes that avoid crosses between closely related individuals in order to maintain a maximal level of heterozygosity in the progeny. However, most outbred stocks such as SW were derived from a small number of mice and are essentially closed colonies originally derived from a very limited gene pool (Lutz et al., 2012). To maintain outbred stocks, unrelated mice are mated by random selection of young breeders, using a random numbers table or computer program. According to the recommendations for small colonies, replacement breeders should be outcrossed every five years (Jackson Laboratory, 2009). In our case, although the colony was maintained under a rotation breeding system, reproductive performance was impaired early. These results suggest that the reproductive performance may be affected earlier than expected, and colony replacement or other strategy should be conducted before to avoid inbreeding conditions.

Reproductive performance in mouse embryo transfer programs depends on genetic background of the recipients and the embryo (Byers et al., 2006; Dorsch et al., 2019), but also on environmental factors. Unpredictable changes in the animal facility variables may also affect the ET outcome, for instance heavy construction noises or sudden noises, humidity, light, among others (Diercks et al., 2010; Rasmussen et al., 2009). However, in the current study no evidence of this kind of changes in the facility was recorded during the 5-year period of the analysis. The quality control program in this SPF facility includes a computerized monitoring system to record and amend any deviation, and the findings of this study were not associated with deviations in this program. In addition, the occurrence of cannibalism in the late phase of the period appeared only in SW but not in B6D2F1 females, while both were maintained under the same conditions. Taking this into account, we suggest that cannibalism would be associated with a failure in the outbreeding maintenance of the colony more than any other reason. This finding should be considered for the outbreeding of other mouse stocks but also for other rodents like rats maintained under similar breeding systems.

When compared with B6D2F1 hybrid females, SW females showed lesser outcomes, even in the first year of evaluation when the results were not affected by the colony aging. F1 hybrid females display an overall hybrid vigor characterized by earlier fertility, larger litter size, higher overall reproductive performance and lower neonatal mortality than parental strains. In addition, they can be easily produced in mouse facilities by crossing inbred strains. All these features and the good maternal ability make them particularly robust and valuable as recipient mothers. In the current study, B6D2F1 females showed larger litter size per female and more pups from transferred embryos than SW outbred stock females. Under these conditions, the use of B6D2F1 hybrid instead of SW females showed better performance, even though the outbred stock colony was recently established.

Litter loss or cannibalism was detected in SW at the end of the analyzed 38-month period in the retrospective analysis, and was confirmed on Year #5 when SW females were compared with B6D2F1. This finding did not occur neither at the beginning of the analyzed period nor on Year #1. Practical observation of litter loss typically implies that no pups are found when the cage is inspected after parturition, and because dead pups are generally eaten, it is commonly assumed that the mothers have killed them. Usually the terms infanticide or cannibalism are used indistinctly to refer to the cause of death. However, infanticide denotes the killing of young by conspecifics; while cannibalism is defined as the eating of conspecifics, including both killing followed by eating or eating already dead conspecifics (Weber and Olsson, 2008). In a standard facility environment, there should be no physical risk of mother starvation, predation or stressor factors that can induce infanticide, and no studies have addressed the problem with maternal infanticide under normal environmental conditions (Weber and Olsson, 2008). Thus, in the current study maternal cannibalism may be a more appropriate term, and probably the cause of litter loss would be associated to fetal or perinatal mortality. For this reason, further examination of uterine environment on SW and B6D2F1 females was performed.

Gross examination of the uterus of SW females showed high incidence of cysts, abnormal implantation sites and hemorrhagic endometrium, a finding that did not occur in B6D2F1 females. Of notice, these abnormalities were found in those recipients that were pregnant and produced cannibalism, but also in those females that produced live pups or in those virgin females that were not mated/transferred. Even though this gross examination was only descriptive and was performed in few animals, the main finding is the occurrence of abnormalities in the uterus of SW females, independently of their reproductive condition. Endometrial cysts have already been associated to impaired fertility in mice (McCallum et al., 2016) and other species (Mir et al., 2013; Tannus and Thun, 1995). We suggest that the impairment on reproductive performance of SW females related with maternal cannibalism could be associated with fetal or perinatal mortality, probably induced by uterine dysfunction. However, other causes of maternal cannibalism should not be excluded and further investigations are required to attend the reason of offspring losses found in the current study.

## Conclusion

This study reports the impairment of the reproductive performance of SW females used as embryo transfer recipients in a research facility during a) a 38-month period, and b) compared with B6D2F1 females in a 5-year interval. The reproductive performance of our SW outbred stock was diminished by an early aging of the colony, which was related to a high incidence of maternal cannibalism. Moreover, when compared to B6D2F1 in a 5-year interval, the hybrid strain showed significantly higher reproductive outcomes. Selection of the female recipient strain should be taken into account when embryo transfer programs are applied as routine in mouse facilities.
